# Implications of Neuroinvasive Bacterial Peptides on Rodents Behaviour and Neurotransmission

**DOI:** 10.3390/pathogens6030027

**Published:** 2017-07-02

**Authors:** Aneela Taj, Nusrat Jamil

**Affiliations:** Department of Microbiology, University of Karachi, Karachi 75270, Pakistan; ain2005_ku@yahoo.com

**Keywords:** neuropathogenic bacteria, bacterial peptides, behaviour, neurotransmission, HPLC-EC

## Abstract

Neuroinvasive microbes are capable of applying their influences on the autonomic nervous system (ANS) of the host followed by the involvement of central nervous system (CNS) by releasing extracellular metabolites that may cause alterations in the biochemical and neurophysiological environment. Consequently synaptic, neuroendocrine, peripheral immune, neuro-immune, and behavioural responses of the host facilitate the progression of infection. The present study was designed to extrapolate the effects of crude and purified extracellular peptides of neuropathogenic bacteria on behavioural responses and neurotransmission of Sprague Dawley (SD) models. *Listeria monocytogenes* (Lm) and *Neisseria meningitides* (Nm) were isolated from the 92 cerebrospinal fluid (CSF) samples collected from mentally compromised patients. *Bacillus cereus* (Bc) and *Clostridium tetani* (Ct) were also included in the study. All bacterial strains were identified by the standard biochemical procedures. Filter sterilized cell free cultural broths (SCFBs) were prepared of different culture media. Behavioural study and neurotransmitter analysis were performed by giving an intraperitoneal (i.p.) injection of each bacterial SCFB to four groups (Test; n = 7) of SD rats, whereas two groups each (Control; n = 7) received a nutrient broth (NB) control and sterile physiological saline control, respectively. Extracellular bioactive peptides of these bacteria were screened and purified. All experiments were repeated using purified bacterial peptides on SD rat cohorts. Our study indicated promising behavioural changes, including fever, swelling, and hind paw paralysis, in SD rat cohorts. Purified bacterial peptides of all bacteria used in the present study elicited marked changes in behaviour through the involvement of the autonomic nervous system. Furthermore, these peptides of meningitis bacteria were found to potently affect the dopaminergic neurotransmission in CNS.

## 1. Introduction

Microbial invasion of the central nervous system (CNS) has been proved to be the most severe and frequently fatal event during the course of many infectious diseases [1,2,3]. A wide variety of neuroinvasive microbes can traverse the CNS haematogenously, transcellulary, paracellulary, and/or by means of infected phagocytes, i.e., Trojan horse mechanisms. Consequently, diversified clinical manifestations, i.e., from viral encephalitis to bacterial meningitis occur [4,5,6,7,8,9,10].

Bacterial meningitis is established to be the most studied amongst the CNS invasions to date [11,12,13]. In recent years, experimental studies have amplified the understanding that meningitis associated brain injury and neuronal death is not mediated simply by the presence of viable bacteria. By contrast, it occurs as a consequence of the host reaction to a wide range of bacterial components and/or molecules [2,14,15]. Lipopolysaccharide (LPS) [16,17,18,19,20], peptidoglycan (PG) [21,22], exotoxins [23], and capsular polysaccharide [24] have been extensively evidenced to enhance neuroinvasion in animal models and/or cell cultures [10], thus serving as the bacterial bioactive molecules. Moreover, attachment of these molecules to host cell triggers the activation of the CNS subsequent to the involvement of the autonomic nervous system (ANS) [15,25]. Consequently, a variety of different host responses, i.e., synaptic, endocrine, immune, and behavioural responses, together with several cellular and biochemical processes, become activated to facilitate the progression of infection [25,26].

Many studies have revealed that alteration in the neurotransmission and consecutive release of certain neurotransmitters may occur when bioactive molecules are either administered peripherally or directly to the brain [18,19,20]. Furthermore, neurochemical studies evidenced that peripheral inflammation, induced by intraperitoneal injection of LPS, resulted in a highly differentiated in vivo noradrenergic neurotransmission in the brain [27] and dopaminergic neurotransmission in the frontal cortex, nucleus accumbens, striatum, amygdala, hippocampus, and hypothalamus [28]. However, earlier literature on endotoxemia and related catecholaminergic neurotransmission revealed quite incompatible data regarding dose-dependent responses to LPS in different brain regions [17].

The present study was designed to understand the effects of crude and purified extracellular peptides of neuropathogenic bacteria on behavioural and neurological responses, i.e., neurotransmission in the CNS of hosts using Sprague Dawley (SD) rat models. This will give an insight of the involvement of autonomic nervous system in the mechanism of the disease production.

## 2. Results

### 2.1. Identification of Etiological Agents from CSF

Bacterial meningitis was found in 21 (22.8%) CSF samples, whereas 71 samples (77.1%) did not yield any bacterial etiology 4.7% of the CSF samples revealed Gram positive rods of *Listeria monocytogenes* (Lm), while Gram-negative kidney shaped *Neisseria meningitides* (Nm) were found in 95.3% of the samples. The reason for the isolation of bacteria from the patient’s CSF was based on the understanding that bacterial pathogens are in their active state during the infection and they may exhibit most of their pathogenic potential in this state. Furthermore, these results confirm the presence of bacterial meningitis in the selected patients.

### 2.2. Physiological Changes

**Figure 1:**
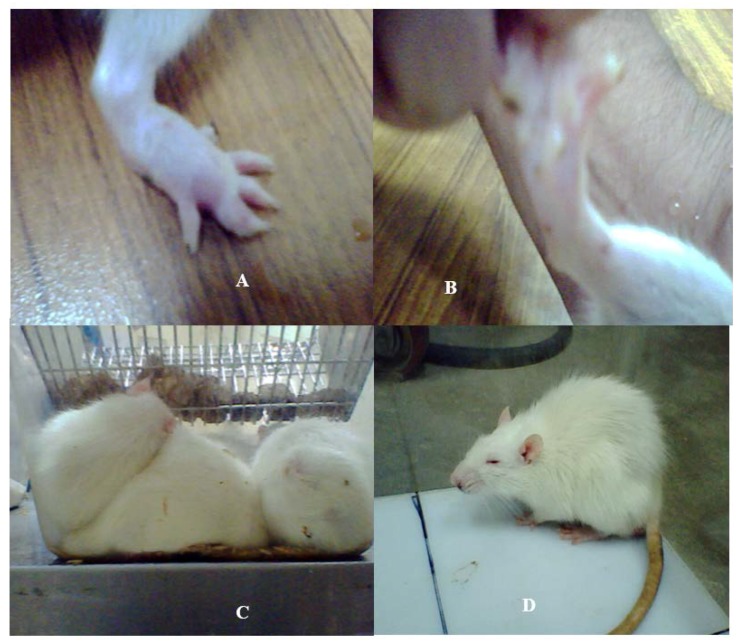
Physiological changes developed in the SD rat model subsequent to the bacterial SCFBs exposure. (**A**) Development of swelling in the SD rat model due to Nm_(SCFB)_ challenge; (**B**) development of hind paw paralysis in the SD rat model due to Ct_(SCFB)_ challenge; (**C**) development of fever and curled body posture in the SD rat model due to Lm_(SCFB)_ challenge; and (**D**) development of piloerection in the SD rat model due to Bc_(SCFB)_ challenge.

This experiment was delineated to evaluate the influence of filter sterilized cell free cultural broth (SCFB) of neuropathogenic bacteria on the physiological responses evoked in SD rats. Animals that received Lm_(SCFB)_ and Bc_(SCFB)_ developed fever within 20 min of injection (Figure 1C,D). In addition to this, Lm_(SCFB)_ caused a curled body posture in rats (Figure 1C) and intense piloerection was observed subsequent to the exposure with Bc_(SCFB)_ (Figure 1D). A hind limb paralysis was observed in rats injected with Ct_(SCFB)_ (Figure 1B). This occurred within 2 min of the challenge. Swelling of the hind paw was observed in rats injected with Nm_(SCFB)_ within 5 min (Figure 1A).

Another set of experiment was conducted to evaluate the influence of neuropathogenic bacterial peptides purified from spent media on the physiological responses evoked in SD rats. It was interesting to note that all rats challenged with Lm_(NB)_ and Lm_(RPMI)_ developed fever. Although fever was not as intense as it was in crude SCFB, the animals were lethargic, movement difficulty, and paw licking were the common features, whereas intense piloerection was observed in SD rat groups injected with the purified peptides of Bc_(NB)_ and Bc_(RPMI)_. Furthermore, rats of Nm_(NB)_ and Nm_(RPMI)_ cohorts revealed drowsiness and were found to be involved in licking the hind paw at the site of injection. Development of insufficient inflammation may be due to the low concentration of injected Nm peptides. In contrast, animals of the Ct_(NB)_ and Ct_(RPMI)_ cohort revealed immobility in all animals of the group, which was due to the development of low intensity muscular spasms. Drowsiness and immobility were the common traits of all test animals.

### 2.3. Home Cage and Open Field Test

**Figure 2:**
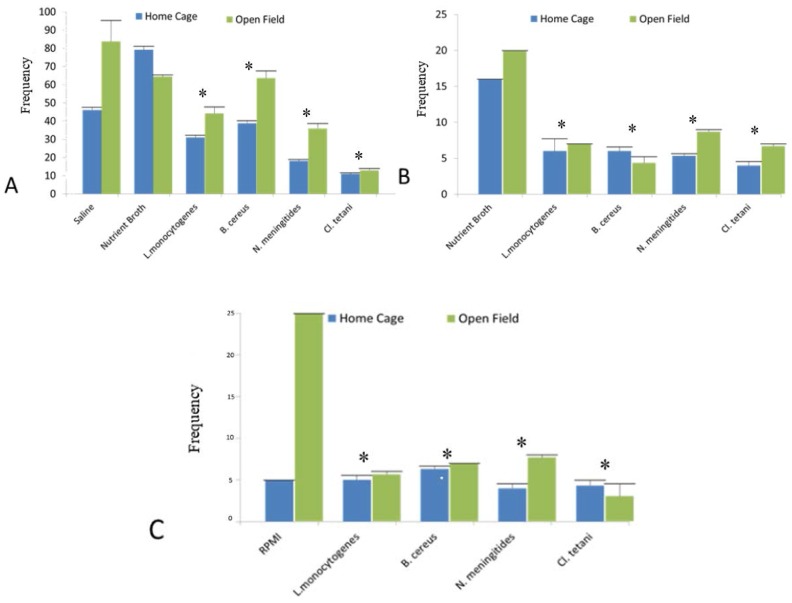
Motor activity profile of the SD rat model post administration of bacterial SCFBs and purified bacterial peptides and controls. (**A**) Changes in the motor activity profile of the SD rat model developed due to Nm_(SCFB)_, Ct_(SCFB)_, Lm_(SCFB)_, and Bc_(SCFB)_ challenge; (**B**) changes in the motor activity profile of the SD rat model developed due to Nm_(NB)_, Ct_(NB)_, Lm_(NB)_, and Bc_(NB)_ peptide challenge; and (**C**) changes in the motor activity profile of the SD rat model developed due to Nm_(RPMI)_, Ct_(RPMI)_, Lm_(RPMI)_, and Bc_(RPMI)_ peptide challenge.

In this experiment, it was evaluated whether the SCFB and pure neuropathogenic bacterial peptides purified from spent media treatment would affect the motor activity of rats in the home cage and open field tests. Two-way ANOVA showed the significant differences in the locomotion (*p* < 0.05) of the rats treated with the Nm_(SCFB)_, Ct_(SCFB)_, Lm_(SCFB)_, and Bc_(SCFB)_ both in home cage and open field tests. Furthermore, characteristic immobility was noted in the SD rat groups challenged with Ct_(SCFB)_ and Lm_(SCFB)_ (Figure 2A).

In addition to this, significant difference (*p* < 0.05) was evaluated using two-way ANOVA in the mobility of the SD rat groups injected with Nm_(NB)_, Ct_(NB)_, Lm_(NB)_, and Bc_(NB)_ peptides (Figure 2B). Likewise, Nm_(RPMI)_, Ct_(RPMI)_, Lm_(RPMI)_, and Bc_(RPMI)_ peptides significantly altered the mobility pattern (*p* < 0.05) of all of the rats of the groups (Figure 2C). All of the results were compared with the respective controls of all SD rat cohorts. Since Figure 2A represents the motor activity profile observed subsequent to the injection of bacterial SCFBs, whereas Figure 2B,C show the motor activity profile of SD rat cohorts challenged with bacterial peptides purified from NB and RPMI, respectively. Therefore, it is significant to mention herein that the basal value and frequency of NB mentioned in Figure 2A is because of plain SCFB, whereas in Figure 2B,C it is because of the peptides purified from the NB and RPMI, respectively (Table 1).

### 2.4. Bioactive Peptides Released by Neuropathogenic Bacteria

Chromatograms of bacterial SCFB were analysed and compared with amino acid standard peaks. All four bacterial SCFBs eluted mixtures of peptides had been confirmed by comparing their elution time with standard amino acid elution time.

### 2.5. Quantification of Neurotransmitters in Response to Crude SCFBs

In this experiment the potential of crude SCFB of neuropathogenic bacteria to affect the neurotransmission in rats was evaluated. Levels of different biogenic amine neurotransmitters were quantified in brains of SD rats of all groups injected with Nm_(SCFB)_, Ct_(SCFB)_, Lm_(SCFB)_, and Bc_(SCFB)_ by taking the standard mean.

**Figure 3:**
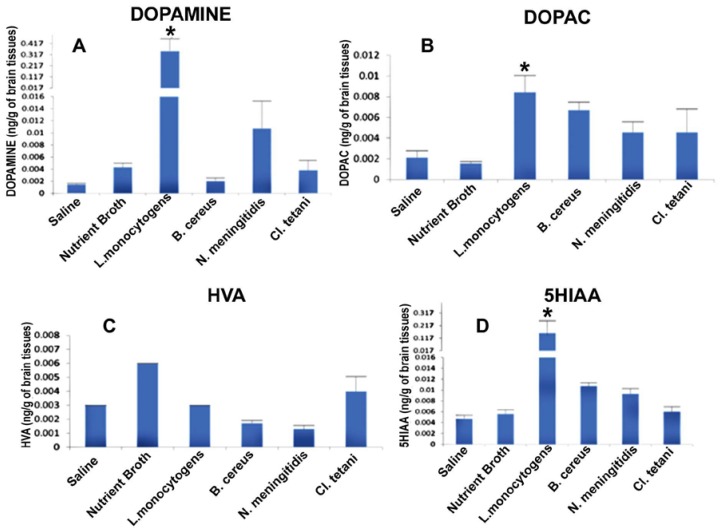
Quantification of neurotransmitters in the whole brains homogenates of SD rat post administration of Nm_(SCFB)_, Ct_(SCFB)_, Lm_(SCFB)_, and Bc_(SCFB)_ and controls. (**A**) The level of dopamine in brain homogenates of control and treated SD rats; (**B**) the level of DOPAC in brain homogenates of control and treated SD rats; (**C**) the level of HVA in brain homogenates of control and treated SD rats; and (**D**) the level of 5HIAA in brain homogenates of control and treated SD rats. Values are expressed as mean ± SEMs * (*p* < 0.05).

It was significant to note that level of dopamine (DA) was significantly enhanced (*p* < 0.05) in the brains of all of the rats, whereas a profound increase was observed due to the challenge of Lm_(SCFB)_ and Nm_(SCFB)_ (Figure 3A). In contrast, the quantity of dihydroxyphenyl acetic acid (DOPAC) was found to be significantly elevated (*p* < 0.05) subsequent to the exposure of Nm_(SCFB)_, Ct_(SCFB)_, Lm_(SCFB)_, and Bc_(SCFB)_ (Figure 3B). In addition to this, 5-hydroxyindol acetic acid (5HIAA) was found increased (*p* < 0.05) in the rats treated with Ct_(SCFB)_ and Lm_(SCFB)_ (Figure 3C). Two-way ANOVA showed a significant increase (*p* < 0.05) in the quantity of homovanillic acid (HVA) in the brain of rats challenged with Nm_(SCFB)_, Ct_(SCFB)_, Lm_(SCFB)_, and Bc_(SCFB)_ (Figure 3D). The chromatograms of test groups were compared with both controls, i.e., saline and control_(SCFB)_.

### 2.6. Quantification of Neurotransmitters in Response to Neuropathogenic Bacterial Peptide

This experiment was outlined to evaluate whether the neuropathogenic bacterial peptides are potent enough to affect the neurotransmission in the SD rat model. Biogenic amine neurotransmitters were quantified in the rat brains challenged with Nm_(NB)_, Ct_(NB)_, Lm_(NB)_, and Bc_(NB)_ peptides by taking the standard mean.

**Figure 4:**
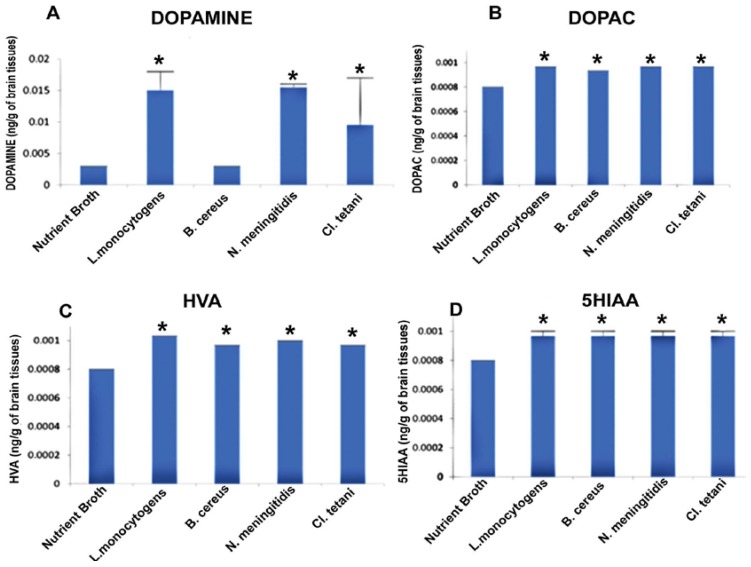
Quantification of neurotransmitters detected in the whole brains homogenates of SD rat post administration of Nm_(NB)_, Ct_(NB)_, Lm_(NB)_, and Bc_(NB)_ and controls. (**A**) The level of dopamine in brain homogenates of control and treated SD rats; (**B**) the level of DOPAC in brain homogenates of control and treated SD rats; (**C**) the level of HVA in brain homogenates of control and treated SD rats; and (**D**) the level of 5HIAA in brain homogenates of control and treated SD rats. Values are expressed as mean ± SEMs * (*p* < 0.05).

Figure 4A describes the significant increase (*p* < 0.05) in the levels of DA subsequent to the exposure to Nm_(NB)_, Ct_(NB)_, and Lm_(NB)_. Levels of DOPAC were significantly elevated (*p* < 0.05) in the brains of all of the rats treated with Nm_(NB)_, Ct_(NB)_, Lm_(NB)_, and Bc_(NB)_ (Figure 4B). Furthermore, noteworthy elevation (*p* < 0.05) was found in the levels of both HVA and 5HIAA in the brains of rats that received Nm_(NB)_, Ct_(NB)_, Lm_(NB)_, and Bc_(NB)_ (Figure 4C,D).

**Figure 5:**
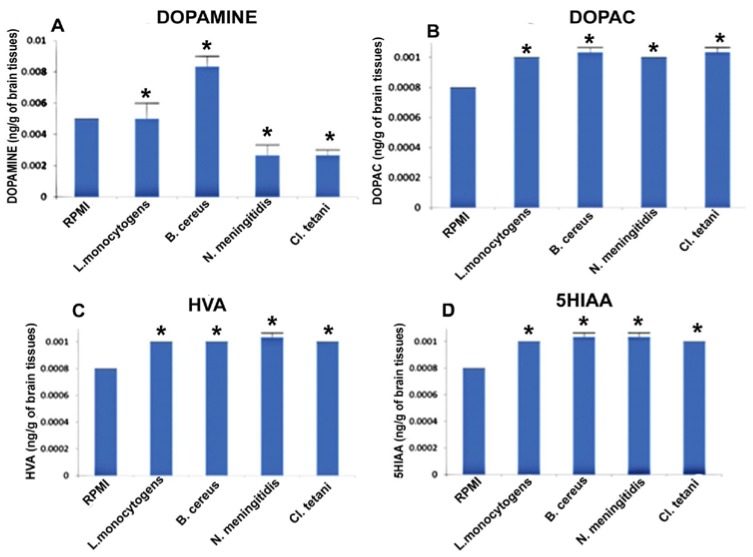
Quantification of neurotransmitters detected in the whole brain homogenates of SD rat post administration of Nm_(RPMI)_, Ct_(RPMI)_, Lm_(RPMI)_, and Bc_(RPMI)_ and controls. (**A**) The level of dopamine in brain homogenates of control and treated SD rats; (**B**) the level of DOPAC in brain homogenates of control and treated SD rats; (**C**) the level of HVA in brain homogenates of control and treated SD rats; and (**D**) the level of 5HIAA in brain homogenates of control and treated SD rats. Values are expressed as mean ± SEMs * (*p* < 0.05).

Figure 5A, on the other hand, revealed the elevation (*p* < 0.05) in the levels of DA in the rats injected with Nm_(RPMI)_, Ct_(RPMI)_, Lm_(RPMI)_, and Bc_(RPMI)_, while Bc_(RPMI)_ was found to be the most potent peptide to affect dopaminergic neurotransmission. In addition to this, profound elevation (*p* < 0.05) was found in the levels of DOPAC, HVA, and 5HIAA in the brains of rats challenged with Nm_(RPMI)_, Ct_(RPMI)_, Lm_(RPMI)_, and Bc_(RPMI)_ peptides (Figure 5B–D).

The chromatograms of test groups were compared with the chromatograms of both Control_(NB)_ and Control_(RPMI)_.

## 3. Discussion

An extensive literature search revealed that LPS [17,18,19,20] and PG [21] have been widely used to mimic infection. Contrary to the previous studies, we focused on the small purified peptides of pathogens grown in different media (NB and RPMI 1640 supplemented with human serum).

The behavioural study of the Sprague Dawley (SD) rat model in response to intraperitoneal (i.p.) injections of SCFB of four neuropathogens reflected a diversified response. It was interesting to note that all animals showed retardation of motor activity (Figure 2) and drowsiness. The recipient cohort of Lm_(SCFB)_, developed fever 20 min later of i.p. injection (Figure 1C), whereas animals in control cohorts (Saline and Control_(SCFB)_) did not reveal any change in their behaviour and body temperature. Thus, it is justified to conclude that extracellular metabolites of Lm are potent enough to induce sluggish behaviour and fever, which is an important component of febrile response. Evidence suggested that microbial exogenous substances (LPS, PG) and viral products can evoke fever in animal models [29]. The present study takes these findings a step further and demonstrated that neuropathogen release certain peptides, which may serve as bioactive molecules and affect the physiology of animals with pronounced fever during the course of disease progression.

Interestingly, pronounced piloerection was observed in rats that received i.p. injections of Bc_(SCFB)_ (1 mL/kg of body weight). This response in test cohort contrary to the control_(SCFB)_ (Figure 3D) was due to the peptides present in SCFBCs. All rats of this cohort showed impaired motor activity (Figure 2), huddled together, were lethargic, and had diarrhoea. The attitude of these animals was very similar to the study of Linthorst et al. [30] who mentioned that i.p. injection of bacterial endotoxin in rats displayed several characteristics reminiscent of sickness, for example, piloerection, curled body posture, and immobility. Our findings are further supported by the study performed by Tavare and Miñano [31] which stated that 15 mg/kg of LPS, (*Escherichia coli*, serotype 0111:B4) induced profound sickness behaviour including piloerection.

Development of intense inflammation in the hind paw was due to i.p. injection in the Nm_(SCFB)_ cohort (Figure 1A) prompted us to assume that Nm_(SCFB)_ had induced inflammatory process. Consequently, phagocytic cells in localized area become active and produced a range of proinflammatory cytokines, i.e., TNFα and IL1β. This was confirmed by the detection of mRNA expressed in cultured peripheral lymphocytes and cultured glial cells (unpublished). These results suggested that the bioactive molecules of Nm seem to be potent enough to provoke the inflammatory cascade. Present results corroborate the findings of Pathan et al. [24] indicated that meningococcal endotoxin triggers an intense inflammatory process, including the production of a range of proinflammatory cytokines when bound with peripheral blood inflammatory cells.

Another acute neurological symptom was observed in the Ct cohort (Figure 1B): i.p. injection of SCFB of this neuropathogen caused immediate paralysis in the hind limb of rats reflecting the presence of toxin in SCFB which affected the localized muscles. These results are in good agreement with the classical symptoms of the tetanus i.e., muscular spasm which is caused by the tetanospasmin. Being an extremely potent neurotoxin, it cleaves vesicle-associated membrane protein synaptobrevin required essentially for the release of glycine and γ-amino butyric acid (GABA). Interneurons inhibiting alpha motor neurons are affected and cause the failure of motor reflex inhibition, resulting in increased muscle tone and rigidity, and sudden and potentially devastating muscle spasms [32].

Development of physiological, neurological, and behavioural symptoms due to SCFB injection primarily uncover a potential link between bacterial extracellular metabolites and central neurotransmission. Therefore, to unveil the underlying association, biogenic amine levels were evaluated in whole brains of SD rat cohorts. Significantly, test cohorts, in contrast to the control cohorts, showed a profound increase in the concentration of dopamine (DA) and its downstream metabolic products, i.e., dihydroxyphenyl acetic acid (DOPAC) and homovanillic acid (HVA). Present findings are, in part, consistent with previous research, which has reported marked changes in catechol and indolamines levels in specific areas of SD rat brains after i.p. LPS injection (*Escherichia coli*, LPS 06:B6) (15μg/kg) [20,21,33,34,35]. It was significant to note that Lm SCFB elicited a pronounced increase in dopamine level (5057 ng/g of brain) among all test cohorts. Thus, establishing a fact that neuropathogenic SCFBs are capable of inducing marked changes in the catecholamine (mainly dopamine) level in the brain (Figure 3). Since dopamine is involved in the control of a wide variety of neuronal functions, like motor coordination, reward driven learning, and cognition, therefore, elevated dopamine seems to be responsible for the impairment of motor coordination and motility. Furthermore, centrally-elevated DA may affect both peripheral and neuro-immune systems by binding and activating the DA receptors present on the surface of immune cells [36,37]. The results of the present study can be further explained by the well-documented evidence that excessive DA undergoes enzymatic degradation subsequent to the re-uptake by pre-synaptic neurons [38]. The detection of intermediate products, i.e., DOPAC and HVA, in all test cohorts confirms the rapid downstream conversion of DA into these precursors. Conversely, further oxidative deamination of DA produce a variety of neurotoxic compounds, i.e., H_2_O_2_ and protein adducts, which leads to the oxidative stress in dopaminergic neurons. Consequently, neuronal damage and/or neurodegeneration of these neurons may occur.

Interestingly, 5HT (4.5 ng/g of brain) was found only in the Bc cohort whereas, in rest of the cohorts, bacterial SCFBs caused elevation in the level of 5HIAA (Figure 3). Hence, this astonishing finding indicated that extracellular metabolites of Bc seems to be capable enough to elevate the level of 5HT. Present results partly match with the study of Linthorst et al. [30] who reported marked increase in rat hippocampal extracellular concentrations of 5HT and 5HIAA due to i.p. injection of LPS. The observed increase in 5HT and its metabolic product 5HIAA can be attributed to the increase in the activity of tryptophan hydroxylase enzyme (TPH), the initial and rate-limiting enzyme in the synthesis of serotonin. Thus, it is possible that Bc_(SCFB)_ instigated the increase in the concentration of 5HT through the subsequent increase in the activity of TPH, which may be activated in order to replenish the stored 5HT following its release. 5HT is mainly involved in memory, so it can be concluded that impaired memory may also be one of the factors seen in bacterial meningitis due to Bc.

The above-mentioned experimental results focused on behavioural and neurotransmitter analysis conducted on the SD rat model in response to crude spent medium lysate of meningitis bacteria. Keeping in view that crude lysate may contain some bioactive molecules either released and/or modified by these pathogens, small peptides were focused. Therefore, subsequent to the peptide purification, all of the experiments were repeated using purified peptides on SD rat cohorts.

It was interesting to note that drowsiness, lethargy, immobility, and licking of the paw were the common features in all cohorts. Furthermore, the Lm cohort developed low-grade fevers subsequent to i.p. injection, whereas intense piloerection was observed in Bc. Animals of the Nm cohort revealed insufficient inflammation, which was attributed to the low concentration of injected Nm peptides (Figure 2B). However, peptides purified from RPMI 1640 supplemented with human serum were found to be more potent to decrease the mobility of all test cohorts (Figure 2C). HPLC-EC analysis of the whole brain of SD rat cohorts separately injected with the peptides of Lm, Nm, Bc, and Ct purified from NB (Figure 4) revealed a profound increase in the concentration of DA in Lm (217.5 ng/g of brain) Nm (224.75 ng/g of brain), and Ct (137.75 ng/g of brain) cohorts respectively. Contrary to NB peptides, no significant increase in the dopamine concentration subsequent to the injection of peptides purified from RPMI 1640 supplemented with human serum (Figure 5) was observed in Lm, Nm, and Ct cohorts. However, peptides of Bc were found competent to cause an increase in DA concentration (116 ng/g of brain) in the brains of all animals of the Bc cohort. Constant elevation of DA due to peptides is contrary to the previous extensive studies in which LPS implication showed either a transitory increase [10,11,12] or a complete decrease [34,35] in DA concentration.

Interestingly, findings of all of the experiments conducted with both crude lysate and purified peptides were significantly superimposed. Therefore, a decline in motor activity in addition to altered behaviour and extremely elevated concentrations of DA in the CNS underpins that manifestations of infection developed in both cohorts were purely attributed to neuropathogenic bacterial peptides. It is significant to highlight that these effects are mainly attributed to the bacterial pathogen affecting CNS. Since the present study comprised of neuroinvasive bacteria based on a literature search that has shown few reports of meningitis caused by *B. cereus* and *Cl. tetani* [39,40], the present study demonstrated that it is not mandatory for bacteria to cross the blood brain barrier (BBB) to affect the neurotransmission in CNS. Entry of bacteria into any part of body has distant effect implications. Physical existence of bacteria may cause the localized infection but their circulating metabolites can intervene in networking of the nervous system through blood circulation and/or by the lymphatic system. Genetic repercussions may occur at the gene expression level including post-transcriptional and post-translational modifications. Sickness behaviour syndrome, along with the activation of physiological and immunological cascades for development of fever and swelling may be due to activation of PNS. Bacterial SCFB did not contain bacteria therefore activation of PNS and localized immune system was purely due to bacterial metabolites, which eventually triggers the involvement of the CNS. Moreover, experiments conducted on the SD rat model further confirmed that small bacterial peptides are potential triggers for activation of the PNS, leading to the CNS.

## 4. Materials and Methods

### 4.1. Chemicals

Unless otherwise stated, all of the analytical grade chemicals and formulated bacteriological media were purchased from Merck, Frankfurter, Darmstadt, Germany. Neurotransmitter standards were obtained from Sigma, St. Louis, Missouri, USA. High-performance liquid chromatography with an electrochemical detection (HPLC-EC) system was acquired from Merck, Frankfurter, Darmstadt, Germany with Chromeleon^®^ 6.8.0 software.

### 4.2. Isolation of Etiological Agents fromCerebrospinalFluid (CSF) Samples

CSF samples were collected from the local diagnostic lab of Karachi with the consent and approval of lab authority. All persons gave their informed consent prior to inclusion in the study. Samples were collected aseptically and kept at −20 °C until further use. For the isolation of etiological agents, 10 μL of each of the CSF sample was streaked separately on different culture media, i.e., nutrient agar, blood agar, and chocolate agar. The experiment was run in duplicate for the isolation of both aerobic and anaerobic bacteria. One set of the media plates were incubated aerobically at 37 °C. For the isolation of the anaerobic etiological agents, another set of the above-mentioned media was incubated at 37 °C in the presence of 5% CO_2_. All plates were initially incubated for 24 h; incubation was then continued up to 48 h. Plates were first observed after 24 h and then after 48 h. Bacteria were identified on the basis of visual colonial characteristics, cellular morphology, and standard biochemical reactions.

### 4.3. Filter-Sterilized Cell Free Cultural Broth (SCFB) Preparation

In order to prepare SCFB, bacterial species i.e., *Bacillus cereus* (Bc), *Clostridium tetani* (Ct), *Listeria monocytogenes* (Lm), and *Neisseria meningitides* (Nm) were grown separately in 100 mL of Nutrient broth (NB) and human serum supplemented RPMI 1640 media (10 mL) for 24 h at 37 °C. Anaerobic conditions were maintained by providing 5% CO_2_. After incubation, each of the cultural broth was centrifuged at 5000 rpm for 10 min; the pellet was discarded and each supernatant was separately air-dried overnight at ambient temperature. Dried broth was scrapped off by adding 2 mL of D/W. Each SCFB was filter-sterilized using a 0.2 micron membrane Millipore filter. SCFBs were marked as Bc, Ct, Lm, and Nm. Blank broths of both of the above-mentioned media were air-dried and filter-sterilized likewise, which served as the control.

### 4.4. Animals

Adult male Sprague Dawley (SD) rats weighing between 220–280 g were used in these experiments. These locally-bred rodents were housed individually with free access to standard rodent diet and water. All animal experiments were conducted in accordance with the policy and standard guidelines for animal experiments of the Institutional Ethics Committee.

### 4.5. Experimental Procedure and Challenge

The study herein reported consisted of two animal models. The first SD rat model was used to measure the effects of crude neuropathogenic bacterial SCFBs on behaviour and neurotransmission. This model was comprised of six groups (7 rats/ group). Four groups were injected intraperitoneally with SCFB (1 mL/kg of body weight) of Bc, Ct, Lm, and Nm. Two control groups received NB control SCFB and sterile pyrogen-free saline (0.9% NaCl), respectively (Table 1).

However, evaluation of neuropathogenic effects of bacterial peptides on the central nervous system (CNS) was carried out in another SD rat model. For the experiment animals were weighed and randomly divided into eight test groups (2 rats/group) and two groups of control. Four test groups were injected with peptides released by each bacterium in nutrient broth, while peptides released by each bacterium in RPMI1640 with human serum were injected to the other four groups. Both control groups, on the other hand, received injections of the peptides extracted from blank NB and RPMI 1640, respectively (Table 1). All animals in this cohort were injected intraperitoneally with 0.5 mg of peptides.

### 4.6. Behavioural Study

Open field and home cage activities were monitored after 45 min of injection for 5 and 10 min, respectively. The open field apparatus used in this study was consisted of a square area 76 × 76 cm with walls of 42 cm height. The floor was divided into 25 equal squares. To determine the open field activity, every animal of both models was placed gently for the first time in the centre square of the field. Locomotion (activities such as walking and running), immobility (absence of movements while the animal is in a lying position), rearing (the animal standing upright on its hind legs mostly against the wall), grooming (shaking, stretching or licking), and the number of squares crossed in the open field were scored for a duration of 5 min. For home cage activity, individual animals were observed in their own cages for movement and grooming for a duration of 10 min.

### 4.7. Extraction of Biogenic Amines

All animals were decapitated 60 min after injection of SCFB and purified bacterial peptides. Brains were quickly removed, washed in sterile pyrogen-free saline(0.9% NaCl), and stored at −80 °C for further analysis. Frozen brain samples were homogenized in 10 volumes of biogenic amine extraction buffer (0.4 M perchloric acid, 0.1% Na-metabisulphite, 0.1% ethylene diamine tetra acetic acid (EDTA), 0.01% cysteine), then centrifuged at 5000 rpm for 10 min. Supernatants were analysed for neurotransmitters concentration by HPLC-EC.

### 4.8. HPLC-EC

HPLC-EC system was consisted of a Dionex ED50 electrochemical detector, a glassy carbon working electrode, 5μm ODS reverse phase, 250 × 4.6 mm, C-18 column, and Chromeleon^®^ 6.8.0 software. The column buffer consisted of 0.1 M NaH_2_PO_4_, 0.1% octyl sodium sulphate, 0.0035% EDTA, and 14% methanol (pH 2.9 adjusted with phosphoric acid).The flow rate of the pump (Dionex Ultimate 3000) was 1 mL/min. The sensitivity of the detector was 1.0 nA, and the potential of the working electrode was 0.6 V. Twenty-five microliters of supernatant was injected into the HPLC-EC system. The peak areas generated from the supernatants were recorded and quantified by comparison with external standards containing 100 ng/mL of dopamine, 3,4-dihydroxyphenylacetic acid (DOPAC), homovanillic acid (HVA), 5-hydroxyindoleacetic acid (5HIAA), and 200 ng/mL of 5-hydroxytryptophan (5HT).

### 4.9. Peptide Extraction

Filter-sterilized cell free cultural broth of bacteria grown in NB and human serum supplemented with RPMI 1640 were selected for the peptides extraction. Twenty-five microliters of each of the bacterial SCFB and control was injected into the HPLC-EC system using 0.1 M KH_2_PO_4_ and acetonitrile (85:15) mobile phases. Peak areas were quantified by comparison with external standards of amino acids. Peptides were identified based on their elution time for purification. Selected peaks were then purified by multiple rounds of HPLC-EC using 0.1 M ammonium acetate and acetonitrile (85:15) buffer. Purity of the peptides was checked using the same buffer. These peptides were freeze-dried and stored at −80 °C till further use.

### 4.10. Sterility Check

Sterility of all the SCFBs and peptides were determined prior to the administration in SD rat models. Ten microliters of each SCFB and peptides were separately streaked on nutrient agar plates. SCFBs and peptides that did not yield any bacterial growth after overnight incubation at 37 °C were used for injecting in the animal model. Sterility was maintained throughout this study. Since the media used for SCFB preparation and peptide extraction were artificial media that do not support the growth of any virus, the possibility of viral contamination in SCFBs and bacterial peptides is eliminated.

### 4.11. Statistics

Statistical data are expressed as mean ± SEMs. The significance (*p* < 0.05) of between-group differences was analysed using the two-way ANOVA tool in SPSS version 15.

## 5. Conclusions

Taken together, the present study highlighted that small peptides released and/or modified by meningitis bacteria are potent in affecting the dopaminergic neurotransmission in the CNS subsequent to affecting the cardinal responses, i.e., behavioural, immunological, and physiological responses. Consequently, these peptides significantly contribute in the progression of infection both in the peripheral and central nervous systems. Since the devastating effects of this disease are found to be associated with the bacterial bioactive molecules, i.e., LPS, PG, and exotoxins, therefore, small bacterial peptides used in this study can be a significant addendum to these known bioactive molecules. Furthermore, the immunogenic potentials of these peptides to trigger both immune and neuro-immune cascades are needed to be addressed (unpublished data).

## Figures and Tables

**Table 1:** Description of the experimental challenges used in the current study.

S. No	Challenge	SD Rat Model Detail
01	SCFBs	Group 1→Bc_(SCFB)_
		Group 2→Ct_(SCFB)_
		Group 3→Lm_(SCFB)_
		Group 4→Nm_(SCFB)_
		Group 5→Control_(SCFB)_
		Group 6→Saline
02	Bacterial peptides purified from spent nutrient broth	Group 1→Bc_(NB)_
		Group 2→Ct_(NB)_
		Group 3→Lm_(NB)_
		Group 4→Nm_(NB)_
		Group 5→Control_(NB)_
03	Bacterial peptides purified from spent RPMI 1640 supplemented with human serum	Group 1→Bc_(RPMI)_
		Group 2→Ct_(RPMI)_
		Group 3→Lm_(RPMI)_
		Group 4→Nm_(RPMI)_
		Group 5→Control_(RPMI)_

## Abbreviations

The following abbreviations are used in this manuscript:

ANS	Autonomic Nervous System
BBB	Blood Brain Barrier
Bc	*B. cereus*
CNS	Central Nervous System
CSF	Cerebrospinal Fluid
Ct	*Cl. tetani*
DOPAC	Dihydroxyphenyl acetic acid
DA	Dopamine
HPLC-EC	High-performance liquid chromatography with electrochemical detection
LPS	Lipopolysaccharide
Lm	*L. monocytogenes*
Nm	*N. meningitidis*
PG	Peptidoglycan
5HT	Serotonin
SD	Sprague Dawley
SCFB	Filter sterilized cell free cultural broth
SCFBs	Filter sterilized cell free cultural broths
